# Synthesis and Cytotoxic Activity of Some New 2,6-Substituted Purines

**DOI:** 10.3390/molecules16075840

**Published:** 2011-07-11

**Authors:** Nageswara Rao Kode, Shashikant Phadtare

**Affiliations:** Division of Basic Pharmaceutical Sciences, College of Pharmacy, Xavier University of Louisiana, 1 Drexel Drive, New Orleans, LA 70125, USA

**Keywords:** Suzuki-Miyaura cross coupling reaction, pyridiniumtribromide bromination, 2,6-substituted purines, cytotoxic activity

## Abstract

A seriesof twenty four acyclic unsaturated 2,6-substututed purines **5a-20b** were synthesized. These compounds were evaluated for cytotoxic activity against NCI-60 DTP human tumor cell line screen at 10µM concentration. N_9_-[(*Z*)-4'-chloro-2'-butenyl-1'-yl]-2,6-dichloropurine(**5a**), N_9_-[4'-chloro-2'-butynyl-1'-yl]-2,6-dichloropurine(**10a**), N_9_-[(*E*)-2',3'-dibromo-4'-chloro-2'-butenyl-1'-yl]-6-methoxypurine(**14**) and N_9_-[4'-chloro-2'-butynyl-1'-yl]-6-(4-methoxyphenyl)-purine(**19**) exhibited highly potent cytotoxic activity with GI_50_ values in the 1–5 µM range for most human tumor cell lines. Other compounds exhibited moderate activity.

## 1. Introduction

**Figure 1:**
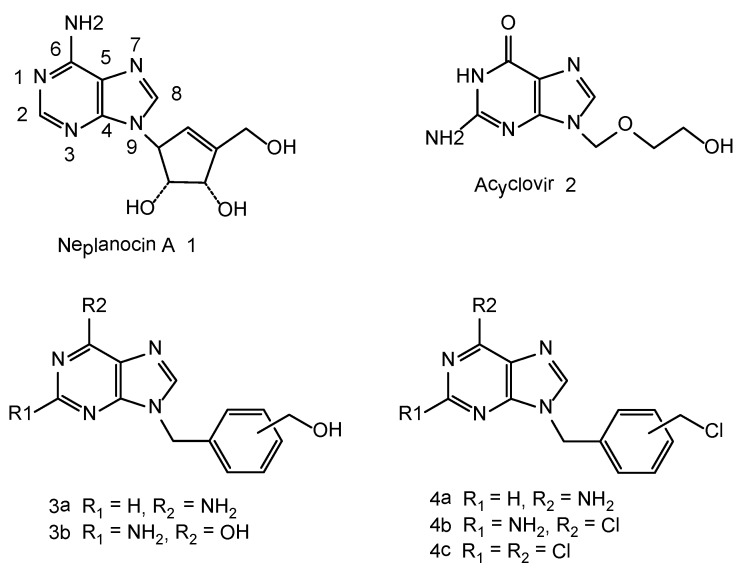
Neplanocin A and aromatic neplanocin-A analogues.

According to WHO report on cancer about 7.6 million people died in the year 2005 and the number is expected to raise to 9 million by the year 2015 and 11.5 million by 2030 [1]. Hence, development of new potent and selective anticancer agents has become one of most intensely pursued goals in drug development around the world*. *Neplanocin A, (**1**, Figure 1) is considered a carbocyclic analogue of a natural nucleoside and has shown potent antitumor and antiviral properties [2,3,4]. As a part of our research program on the synthesis of anti-cancer agents, we have synthesized some aromatic neplanocin-A analogues like **3a-3b, 4a-c** [5,6,7]. 

The N_9_-hydroxymethyl analogues of adenine, guanine and 2,6-diaminopurine related to **3a-3b** did not exhibit any anticancer activity, however, their N_9_-chloromethyl arylpurine intermediates, related to **4a-4c **(Figure 1), were found to be potent *in vitro* growth inhibitors of several human tumor cell lines. These results prompted us to consider purines with an unsaturated N_9_-linker that has been terminated with a chloromethyl group. 

In the synthesis of anti-cancer purine compounds, many times either the purine base is modified or the sugar moiety is modified or replaced with a non-sugar linker or sometimes all these changes have been done by researchers simultaneously in an attempt to make an active compound. We have followed a very similar path in the present work.

*Purine base selection*: 2,6-Dichloropurine is selected where the chlorines are expected to serve as powerful electron withdrawing centers on the purine ring. 2-Chloro-6-methoxypurine is expected to serve a double role, with electron withdrawing and electron donating centers on the purine ring. The 6-methoxy group was selected for its electron donating nature to the purine ring. 6-(4-Methoxy)phenyl- and 6-(4-fluoro)phenyl-substituted purines were selected to significantly alter the purine base properties and to improve the lipophilicity. It is interesting to note that purines with those substitution patterns were also reported to elicit wide range of anti-viral and anti-cancer activities. 6-Methoxypurine arabinoside was reported as a potent inhibitor of Varicella-Zoster virus [8]. 6-Methoxy group-containing Nelarabine and the 2-chloro group-containing compound Clofarabine elicit anti-cancer activities [9]. Further, 6-(4-methoxphenyl)purine and 6-(4-fluorophenyl)purine ribonucleosides were reported to elicit significant cytostatic activity [10].

**Figure 2:**
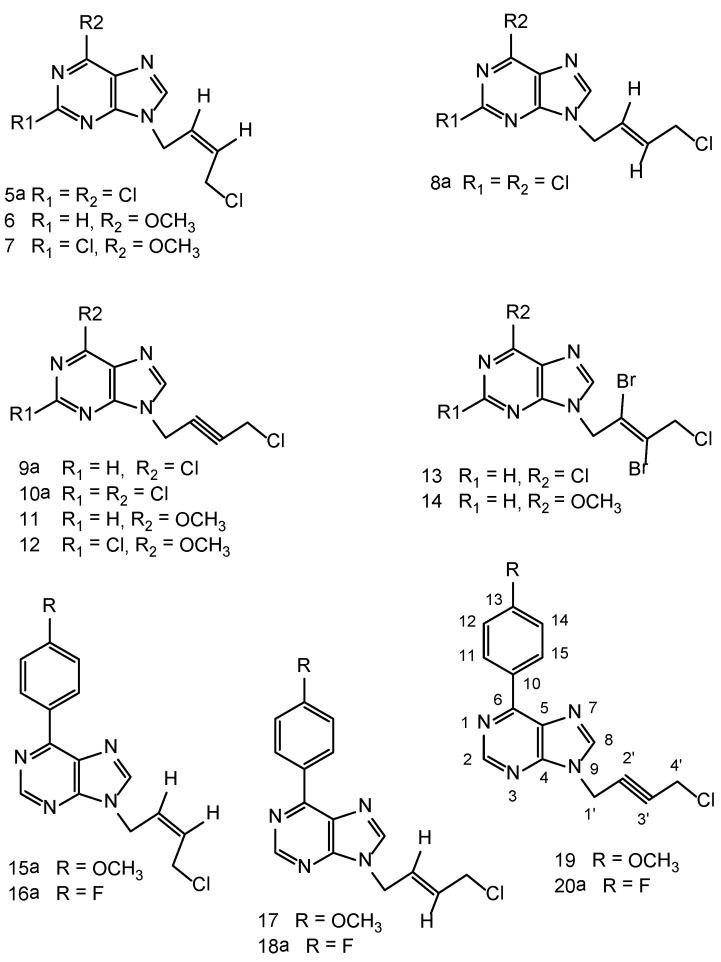
Purines with acyclic unsaturated linkers.

*Linker selection*: We chose linkers like *cis*-1,4-dichlorobutene, *trans*-1,4-dichlorobutene and 1,4-dichlorobutyne. All these linkers are acyclic five carbon length open chain liners analogous to the linker of acyclovir, with some degree of unsaturation. All these liners are common for each of the above purine bases selected, like 2,6-dichloropurine, 6-methoxyurine, 2-chloro-6-methoxypurine, 6-(4-methoxyphenyl) purine and 6-(4-fluorophenyl)purine. Reaction of each of these purine bases with *cis*-a 1,4-dichlorobutene linker furnishes N_9_ substituted purines with methylchloromethyl-*cis*-butene units, e.g., compounds **5a**, **6**, **7**, **15a **and **16a**. Reaction with the *trans*-1,4-dichlorobutene linker furnishes N_9_ substituted purines with methylchloromethyl-*trans*-butene units, e.g., compounds **8a**, **17 and 18a**. Reactionwith1,4-dichlorobutyne is expected to furnish N_9_ substituted purines with methylchloro-methyl-butyne moieties, e.g. compounds **9a**, **10a**, **11**, **12**, **19 **and **20a**. Compounds **13 **and **14 **represent vinylic dibromides, a new class of purines, which were also synthesized in this work to assess their cytotoxic activity. This plan gives an opportunity for us to assess the cytotoxicity for a group of purine compounds (Figure 2) and to understand how the activity is changing for a given linker with a change on the substitution pattern on the purine ring.

Here, we have focused primarily on the synthesis of N_9_ substituted purines because they were found to be more active when compared to N_7_ isomers at our laboratory. Furthermore the N_7_ isomers are expected to be minor products in the synthesis.

## 2. Results and Discussion

### 2.1. Chemistry

**Scheme 1:**
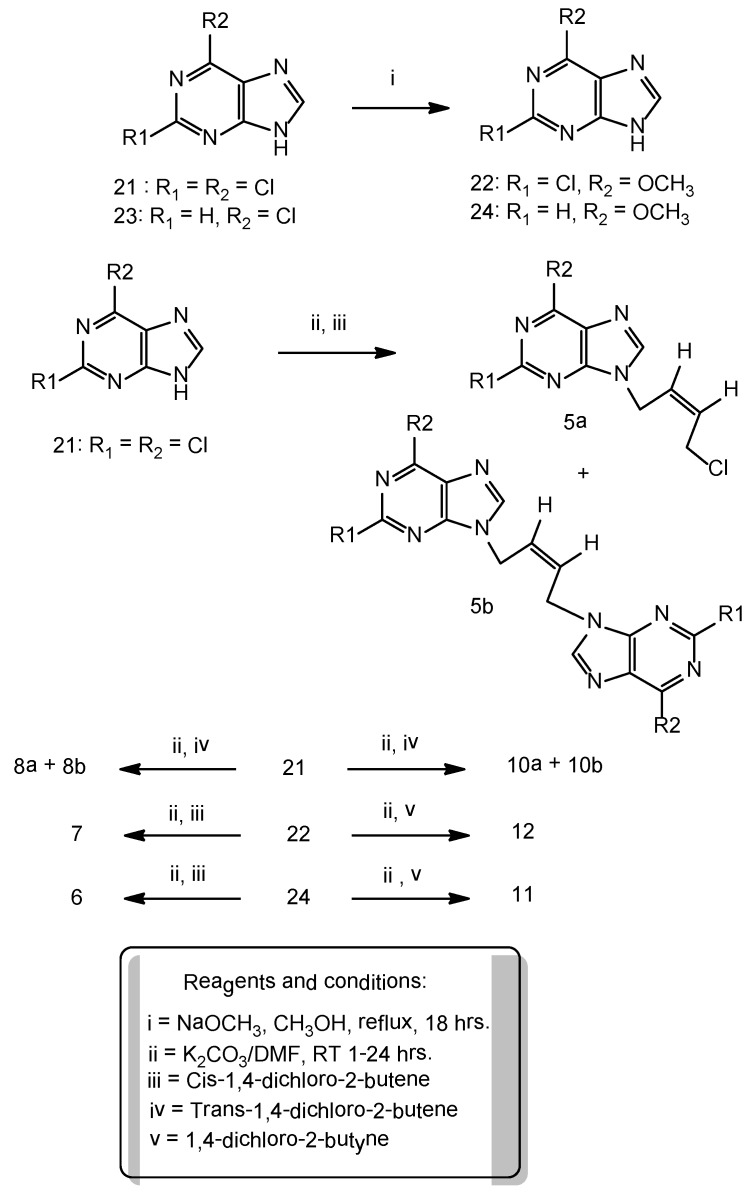
Synthesis of N_9_-alkylated compounds.

**Scheme 2:**
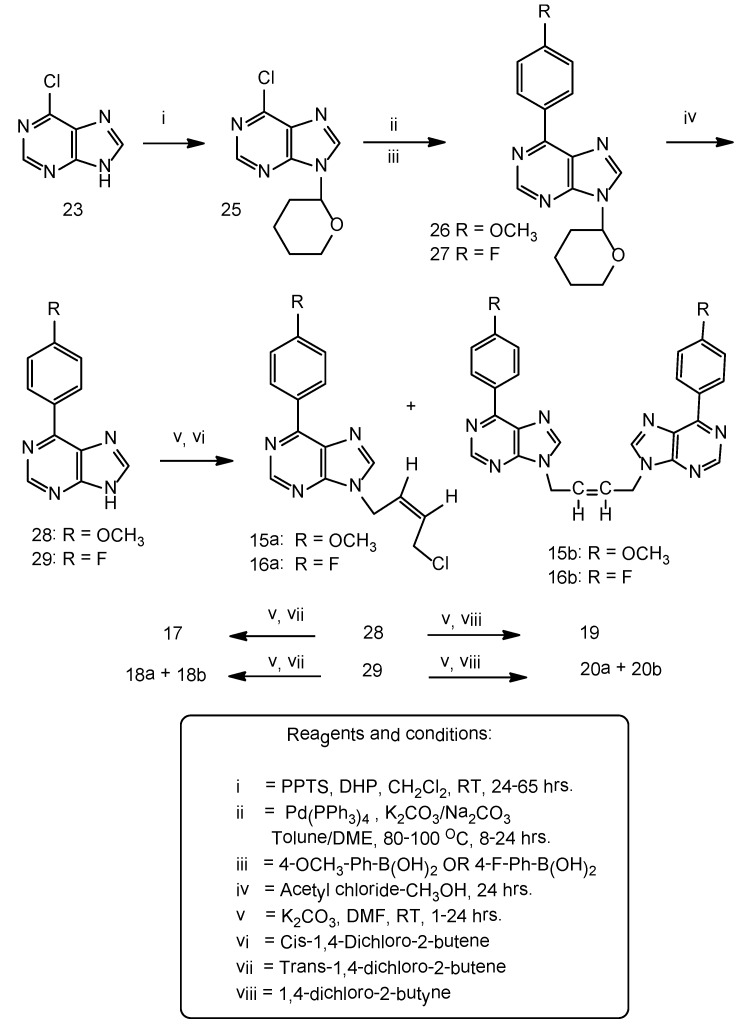
Synthesis of 6-(4'-methoxyphenyl) and 6-(4'-fluorophenyl)-purines.

The N_9_-alkylated compounds **5a-20b** were prepared by the direct alkylation approach on the appropriately substituted purine bases in presence of K_2_CO_3_ in dimethyl formamide (DMF) medium (Scheme 1 and Scheme 2). A 1-3 fold excess of the alkylating agent and anhydrous potassium carbonate were employed for one equivalent of the purine base taken to isolate N_9_-purine isomers as the major product in moderate to good yields. 

**Figure 3:**
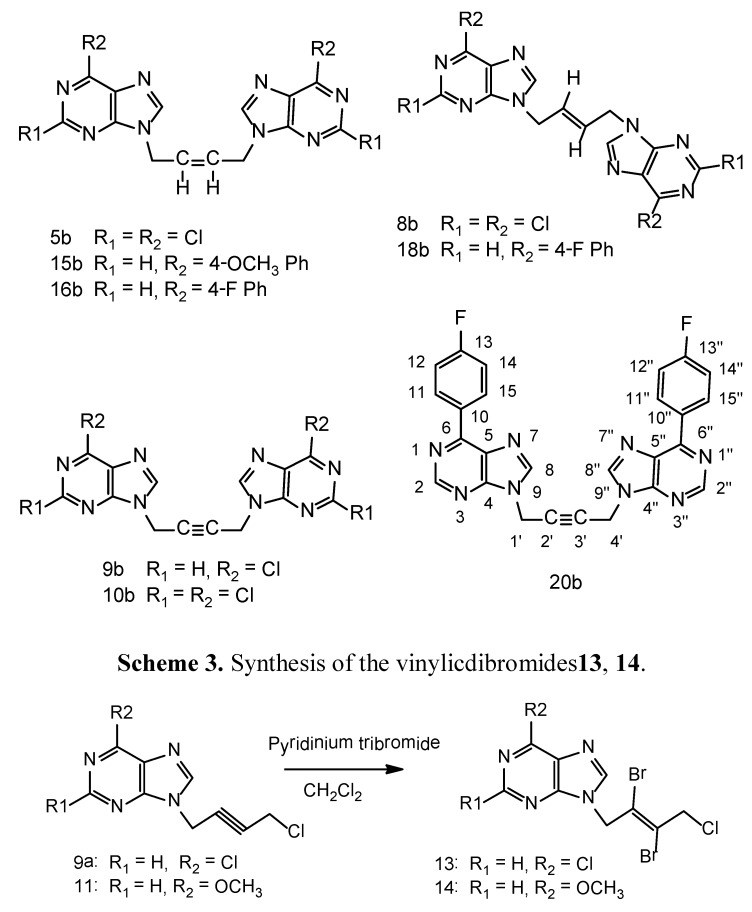
Dimers.

A ten equivalent excess is not required as previously reported [12]. Minor dimeric products (Figure 3) have been isolated whenever formed during the synthesis for each linker. Increasing the reaction time and the molar ratio of the potassium carbonate favors the higher yields of the dimeric products. The UV maxima for N7 isomers were 10–15 nm higher (275–320 nm) than the N_9_-isomers (265–310 nm) [13].

**Scheme 3:**
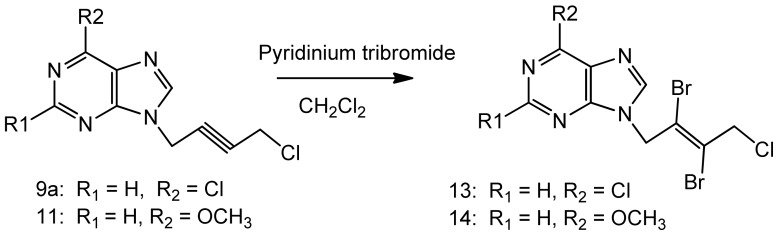
Synthesis of the vinylicdibromides **13**, **14**.

Minor modifications were made to the Suzuki-Miyaura cross coupling procedure [10,14]. Reaction of appropriate phenylboronic acid with 9-(tetrahydropyran-2-yl)-6-chloropurine under Suzuki-Miyaura cross coupling methodology afforded 6-(4'-methoxyphenyl), 6-(4'-fluorophenyl)-purines (Scheme 2). The reported procedure of pyridiniumtribromide bromination of the acetyleneic compounds [11] was adopted with minor modifications to furnish the vinylicdibromides **13**, **14** (Scheme 3).

Acetyl chloride-mediated THP protection and deprotection of hydroxyl functional groups on a wide range of aliphatic and aromatic systems has been reported [15]. We have extended the concept to 6-chloropurine and found acetyl chloride to be a versatile clean inexpensive deprotective agent in methanol medium for the THP removal. No side products are found on the procedure we described, although Dowex 50W X 8 works [10]. During the protection of the NH of 6-chloropurine, acetyl chloride was found to form one side product, possibly an N-acetyl derivative, still the % yield was about 70 after column purification. The pyridinium-*p*-toluenesulfonate (PPTS) catalyzed THP protection of 6-chloropurine was found to be relatively very clean, no major side products formed and the yield was about 90%. Hence we chose PPTS catalyst for the THP protection of 6-chloropurine.

Reaction of sodium methoxide with 6-chloropurine and 2,6-dichloropurine in methanol medium furnished 6-methoxypurine and 2-chloro-6-methoxypurine.respectively. Further reaction of these purine bases with various alkenyl and akynyl linkers as explained above furnished the target compounds **5**a-**20b**. The structures of all the purines **5a**-**20b** were confirmed by ^1^H-NMR, ^13^C-NMR, LC-MS and satisfactory elemental (C, H, N) analysis within ± 0.4% of theoretical values. 

### 2.2. Cytotoxicity

Compounds **5a**, **5b**, **6**, **7**, **8a**, **8b**, **9a**, **9b**, **10a**, **10b**, **11**, **12**, **13**, **14**, **15a**, **15b**, **16a**, **16b**, **17**, **19**, **20 **and **20b** were initially tested at 10 µM concentration (Table 1). 

**Table 1:** Cytotoxicity Data.

Cell Line	5a	5b	8b	10a	10b	14	17	19
**Leukemia**								
**CCRF-CEM**	–45	–73	18	–76	–62	–39	63	19
**HL** **-60(TB)**	–71	–70	–3.0	–70	–58	47	65	49
**K** **-562**	–63	–25	17	–67	–13	8	67	58
**MOLT** **-4**	–69	–55	81	–63	1.0	19	--	5.0
**RPMI-8226**	–53	–61	–17	–76	–57	–25	52	35
**SR**	–76	–48	–14	–76	–53	8.0	--	--
**Non-Small Cell Lung Cancer**								
**A549/ATCC**	–76	–7.0	72	–77	9.0	9.0	81	60
**EKVX**	–91	–68	87	–72	–39	114	108	115
**HOP** **-62**	–72	23	94	–82	62	16	99	77
**HOP-92**	–93	–34	–13	–92	–88	--	38	9.0
**NCI-H226**	–74	–59	94	–81	6.0	--	89	57
**NCI-H23**	–78	9.0	95	–70	39	15	64	59
**NCI-H322M**	–97	21	95	–100	47	79	100	92
**NCI-H460**	–63	4.0	84	–66	13	–41	67	68
**NCI-H522**	–85	–77	28	–89	–71	–72	49	49
**Colon Cancer**								
**COLO 205**	–65	–41	63	–77	–27	–82	87	80
**HCC-2998**	–87	--	--	–85	--	90	91	85
**HCT-116**	–80	–71	33	–100	–35	–82	46	52
**HCT** **-15**	–79	–44	50	–90	–66	32	61	50
**HT-29**	–61	–66	48	–70	–44	–57	85	94
**KM-12**	–86	–69	79	–73	–45	–6	85	73
**SW-620**	–87	–52	45	–89	–45	–74	61	49
**CNS Cancer**								
**SF-268**	–83	–2.0	62	–74	–38	12	88	63
**SF-295**	–92	–33	60	–4	6.0	97	109	95
**SF-539**	–90	–53	47	–93	–66	12	76	92
**SNB-19**	–89	10	75	–99	12	90	88	72
**SNB-75**	–92	–26	65	–97	8.0	47	128	94
**U251**	–72	–56	57	–96	–76	–72	64	29
**Melanoma**								
**LOX IMVI**	–83	–80	34	–89	–83	–87	54	–32
**MALME** **-3M**	–73	–72	111	–70	–75	–7	62	20
**M14**	–85	–33	101	–93	8.0	–25	71	70
**MDA-MB-435**	–90	–67	98	–86	4.0	4	58	40
**SK-MEL-2**	–77	24	96	–76	65	52	69	53
**SK-MEL-28**	–96	–19	99	–98	–26		98	87
**SK-MEL-5**	–91	–82	69	–96	–67	54	86	62
**UACC-257**	–90	--	--	–91	--	–41	81	59
**UACC-62**	–96	–73	79	–92	–12	57	77	51
**Ovarian cancer**								
**IGROV1**	–96	–82	18	–93	–75	12	94	46
**OVCAR-3**	–97	–99	61	–93	–91	–94	78	53
**OVCAR-4**	–86	–89	95	–88	–71	–47	53	51
**OVCAR-5**	–85	–9.0	112	–86	–3.0	62	114	97
**OVCAR-8**	–83	–57	8.0	–84	–35	1	67	65
**NCI/ADR-Res**	–56	30	99	–57	60	–40	77	56
**SK-OV-3**	–98	33	103	–99	88	148	111	86
**Renal cancer**								
**786-0**	–92	–9.0	93	–99	8.0	8.0	70	36
**A498**	–92	–18	90	–89	--	95	68	56
**ACHN**	–89	–94	90	–99	–53	15	59	53
**CAKI-1**	–95	–97	–60	–88	–96	96	71	62
**RXF 393**	--	--	--	--	3.0	70	8.0	30
**SN12C**	–84	–11	96	–87	--	11	84	46
**TK-10**	–86	–45	80	–93	–32	51	86	70
**UO-31**	–95	--	6.0	–98	--	57	60	–87
**Prostate cancer**								
**PC-3**	–93	–58	67	–98	–72	46	84	57
**DU-145**	–99	–98	44	–100	–87	37	82	88
**Breast cancer**								
**MCF-7**	–49	–78	38	–52	–23	0.0	40	36
**MDA-MB-231/ATCC**	–90	–68	96	–89	–53	42	89	70
**HS578T**	–43	–42	46	–44	--	24	131	107
**BT549**	–94	--	–33	–93	--	70	54	50
**T-47D**	–54	–44	38	–62	–21	1.0	68	53
**MDA-MB-468**	–72	--		–61	--	17	–4.0	21
**MCF-7**	–49	–78	38	–52	–23	0.0	40	36

* Where the number 100 = control growth, 0 = 100% inhibition, –100 = total cell kill. Compounds **5, 6, 7a, 10, 11, 14a-b, 15a-b, 17a-b, 19a-b **were not active at 10 µM concentration. * Growth % for one dose testing at 10 µM concentration.

Out of these compounds **6**, **7**, **8a**, **11**, **12**, **15a**, **15b**, **16a**, **16b**, **20a**, **20b **exhibited growth %in the range of 70–100 plus and hence may be considered inactive. Table 1 summarizes the single dose 10 µM test results for the active compounds. Compounds **5a**, **5b**, **8b**, **10a**, **10b **and **14** elicited significant cytotoxicity on almost all the cell lines such as leukemia, non-small cell lung cancer, colon cancer, CNS cancer, melanoma, ovarian cancer, renal cancer, prostate cancer and breast cancer. Under the same single dose testing, compound **17** elicited cytotoxicity to MDA-MB-468 breast cancer cell line while compound **19 **elicited cytotoxicity to LOXIMVI melanoma and UO-32 renal cancer cell lines. Table 2 represent the five dose testing results GI_50_ and LC_50_for compounds **5a**, **10a**, **14 **and **19**. Compound **5a** was found very active with GI_50_ values 1–2 µM for almost all the cell lines. Compound **10a** was also found very active with GI_50_values under 2 µM for many cell lines. For leukemia HL-60TB and melanoma UACC-62 the GI_50_ values are 3.5 and 3.7 µM respectively. Compound **14 **displayed impressive activity, with GI_50_ values of 2–3 µM for leukemia, melanoma, renal cancer and breast cancer. It also elicited significant activity on non-small cell lung cancer, colon cancer and CNS cancer. Compound **19** exhibited striking activity, with GI_50_ values of 2–4 µM for breast cancer and 2–8 µM for leukemia, colon, renal and prostate cell lines. Replacing the chlorine at 6-position in compound **5a** with a methoxy group results in compound **11**. Similarly replacing6-chlorine in compound **5a** with a methoxy group results in compound **12**. These changes resulted in a total loss of cytotoxicity. When the N-9 *cis*-butene stereochemistry in **5a** is changed to a *trans* form as in **8a** also resulted in the loss of activity, although the corresponding dimer **8b **with a *trans* stereochemistry elicited good cytotoxicity. 

On the 6-phenyl substituted compounds, only **19** elicited good activity and all other compounds were inactive. The reported procedure [11] was employed to transform the triple bond compounds **9a **and **11 **in to the corresponding vinylicdibromides**13** and **14** (Scheme 2). Indeed one of the vinylic dibromide with a 6-methoxy substituent, **14**, was found very active for leukemia, melanoma, renal cancer, breast cancer (GI 50 value 2–3 µM) and significant activity on all other cancers. The other vinylic dibromide with a 6-chlorine group **13** did not elicit any cytotoxicity. Compounds **18a** and **18b** were not tested.

**Table 2:** GI_50_ and LC_50_ data. Units: µM.

Compound	5a		10a		14		19	
Cell Line	GI 50	LC 50	GI 50	LC 50	GI 50	LC 50	GI 50	LC 50
**Leukemia**								
**CCRF-CEM**	**0.19**	**0.78**	**0.21**	**0.93**	**2.73**	**>100**	**3.84**	**>100**
**HL-60(TB)**	**0.33**	**>100**	**3.50**	**4.89**	**2.03**	**>100**	**3.06**	**>100**
**K-562**	**0.23**	**>100**	**0.22**	**4.89**	**3.29**	**>100**	**8.06**	**>100**
**MOLT-4**	**0.23**	**>100**	**0.50**	**>100**	**2.91**	**>100**	**2.68**	**>100**
**RPMI-8226**	**0.64**	**>100**	**0.23**	**>100**	**3.19**	**>100**	**3.46**	**>100**
**SR**	**--**	**--**	**--**	**--**	**2.73**	**>100**	**5.24**	**>100**
**Non-Small Cell Lung Cancer**								
**A549/ATCC**	**0.37**	**>100**	**0.54**	**>100**	**10.80**	**62.50**	**7.40**	**>100**
**EKVX**	**1.54**	**7.18**	**2.01**	**8.83**	**16.30**	**55.10**	**12.80**	**68.70**
**HOP-62**	**0.19**	**7.18**	**0.23**	**0.94**	**3.90**	**37.50**	**1.79**	**61.10**
**HOP-92**	**0.20**	**3.41**	**0.73**	**5.70**	**4.08**	**45.30**	**2.75**	**49.80**
**NCI-H226**	**1.30**	**5.10**	**1.61**	**5.70**	**2.82**	**30.70**	**4.45**	**>100**
**NCI-H23**	**0.22**	**5.10**	**0.19**	**0.84**	**3.16**	**39.40**	**7.83**	**50.50**
**NCI-H322M**	**1.64**	**5.68**	**1.63**	**5.59**	**12.40**	**50.20**	**12.50**	**52.30**
**NCI-H460**	**0.20**	**0.67**	**0.19**	**0.78**	**--**	**--**	**3.27**	**45.50**
**NCI-H522**	**1.01**	**7.51**	**2.41**	**29.0**	**0.88**	**5.27**	**3.09**	**55.10**
**Colon Cancer**								
**COLO 205**	**0.20**	**0.69**	**0.19**	**0.67**	**1.73**	**5.83**	**3.51**	**41.60**
**HCC-2998**	**0.20**	**0.71**	**0.27**	**0.72**	**17.20**	**56.90**	**17.60**	**57.20**
**HCT-116**	**0.18**	**0.71**	**0.20**	**0.76**	**1.73**	**7.05**	**3.50**	**41.30**
**HCT-15**	**0.17**	**0.65**	**0.18**	**0.73**	**1.84**	**9.39**	**4.06**	**54.90**
**HT-29**	**0.26**	**>100**	**0.23**	**>100**	**1.63**	**8.41**	**7.12**	**71.70**
**KM-12**	**0.22**	**0.80**	**0.36**	**3.73**	**3.54**	**37.60**	**4.84**	**45.20**
**SW-620**	**0.20**	**0.73**	**0.22**	**3.73**	**--**	**--**	**2.99**	**42.40**
**CNS Cancer**								
**SF-268**	**0.20**	**0.62**	**0.19**	**0.67**	**1.96**	**20.30**	**1.66**	**10.90**
**SF-295**	**1.29**	**6.97**	**1.60**	**0.70**	**10.60**	**51.60**	**2.35**	**40.30**
**SF-539**	**0.12**	**0.53**	**1.03**	**4.92**	**2.17**	**28.00**	**6.48**	**52.70**
**SNB-19**	**0.91**	**4.72**	**0.57**	**4.46**	**12.10**	**49.40**	**4.87**	**44.20**
**SNB-75**	**0.18**	**0.68**	**0.20**	**0.73**	**2.01**	**29.60**	**1.64**	**18.20**
**U251**	**0.18**	**0.57**	**0.18**	**0.62**	**1.74**	**7.62**	**1.96**	**>100**
**Melanoma**								
**LOX IMVI**	**0.17**	**0.62**	**0.17**	**0.65**	**2.79**	**31.30**	**1.77**	**7.01**
**MALME-3M**	**0.22**	**1.32**	**0.22**	**2.67**	**1.49**	**6.89**	**2.58**	**60.10**
**M14**	**0.42**	**6.76**	**0.39**	**6.01**	**1.82**	**9.43**	**4.55**	**45.90**
**MDA-MB-435**	**0.21**	**1.71**	**0.32**	**6.14**	**2.94**	**39.20**	**2.71**	**42.80**
**SK-MEL-2**	**--**	**--**	**--**	**--**	**--**	**--**	**--**	**--**
**SK-MEL-28**	**0.20**	**0.62**	**0.20**	**0.66**	**2.62**	**30.00**	**9.87**	**47.50**
**SK-MEL-5**	**0.28**	**3.53**	**0.28**	**3.82**	**2.19**	**18.90**	**3.16**	**36.20**
**UACC-257**	**0.24**	**1.34**	**0.21**	**0.90**	**0.78**	**21.00**	**5.74**	**64.70**
**UACC-62**	**0.22**	**0.83**	**3.66**	**4.33**	**2.65**	**34.60**	**3.32**	**42.30**
**Ovarian Cancer**								
**IGROV1**	**0.21**	**0.79**	**0.21**	**0.74**	**1.77**	**18.20**	**--**	**--**
**OVCAR-3**	**0.20**	**0.59**	**0.19**	**0.61**	**1.77**	**5.86**	**1.93**	**22.30**
**OVCAR-4**	**0.39**	**4.52**	**0.41**	**>100**	**1.61**	**5.67**	**2.66**	**36.20**
**OVCAR-5**	**0.20**	**0.72**	**0.40**	**0.95**	**3.83**	**39.20**	**3.25**	**37.30**
**OVCAR-8**	**0.24**	**2.80**	**0.25**	**4.51**	**3.01**	**28.50**	**11.60**	**>100**
**NCI/ADR-Res**	**0.21**	**2.80**	**0.24**	**0.90**	**6.12**	**44.20**	**2.72**	**>100**
**SK-OV-3**	**1.69**	**5.64**	**1.74**	**5.72**	**14.80**	**52.90**	**10.50**	**49.00**
**Renal Cancer**								
**786-0**	**0.45**	**6.08**	**0.75**	**6.66**	**3.38**	**33.90**	**1.86**	**8.33**
**A498**	**1.80**	**6.35**	**1.80**	**6.64**	**--**	**--**	**2.15**	**47.50**
**ACHN**	**0.55**	**4.32**	**1.52**	**5.43**	**2.14**	**13.50**	**3.63**	**42.10**
**CAKI-1**	**0.19**	**0.74**	**0.20**	**0.75**	**1.87**	**8.00**	**1.47**	**6.56**
**RXF 393**	**--**	**--**	**--**	**--**	**1.64**	**5.69**	**2.29**	**25.20**
**SN12C**	**0.26**	**1.75**	**0.37**	**6.16**	**2.44**	**31.00**	**2.26**	**29.00**
**TK-10**	**1.82**	**6.34**	**1.56**	**5.98**	**2.54**	**26.90**	**7.29**	**67.50**
**Prostate Cancer**								
**PC-3**	**0.18**	**0.62**	**0.20**	**0.663**	**9.12**	**55.90**	**6.82**	**74.90**
**DU-145**	**0.20**	**0.62**	**0.18**	**0.59**	**5.31**	**42.70**	**4.74**	**45.40**
**Breast Cancer**								
**MCF-7**	**0.16**	**0.82**	**0.16**	**0.77**	**1.43**	**6.54**	**2.48**	**44.10**
**MDA-MB-231/ATCC**	**0.18**	**0.62**	**0.18**	**0.70**	**2.40**	**26.10**	**2.32**	**39.20**
**HS 578T**	**0.26**	**>100**	**0.28**	**>100**	**--**	**--**	**3.80**	**>100**
**BT549**	**0.21**	**0.74**	**0.50**	**4.21**	**--**	**--**	**2.98**	**38.80**
**T-47D**	**0.23**	**0.74**	**0.23**	**0.48**	**2.37**	**78.50**	**2.89**	**56.80**
**MDA-MB-468**	**0.21**	**0.74**	**0.21**	**>100**	**1.56**	**6.91**	**2.37**	**44.80**
**MCF-7**	**0.16**	**0.82**	**0.16**	**0.77**	**1.43**	**6.54**	**2.48**	**44.10**

### 2.3. Pharmacology

A total of 60 human cell lines, derived from nine cancer types (leukemia, lung, colon, brain, melanoma, ovarian, renal, prostate, breast) formed the basis of this NCI-60 DTP human tumor cell line screen [16,17]. The tumor cells were cultured in RPMI1640 medium supplemented with 5% fetal bovine serum and 2 mM L-glutamine. The tumor cells were inoculated in to 96-well microtiter plates, 100 µL at plating densities ranging from 5,000 to 40,000 cells/well depending on the doubling time of the individual cell lines [16,17,18,19].After this cell inoculation, the microtiter plates are incubated at 37 °C, 5% CO_2_, 95% air and 100% relative humidity for 24 h. Two plates of each cell line are fixed in situ with TCA to represent a measure of the cell population (T0)before adding the target compounds. The target compounds are dissolved in DMSO and diluted in the test medium to obtain the desired concentration. 100 µL of each of the test compound solution is now added to the above appropriate cell line microtiter wells and incubated for 48 h at 37 °C, 5% CO_2_, 95% air and 100% relative humidity. A sulforhodamine B (SRB) protein assay was used to estimate cell viability or growth. The cytotoxic effects were evaluated and the assay results and dose response parameters calculated as previously described [17,20,21,22]. At the present time at NCI the target compounds were tested initially at a single dose at 10 µM concentration and those promising target compounds are further tested at five dose testing concentrations 0.01, 0.1, 1.0, 10, 100 µM.

Concentration parameters GI_50_, TGI and LC_50_: The NCI re-named the IC_50_ as GI_50_. GI_50_ value represents the concentration of the target compound that causes 50% growth inhibition, that is derived from the formula 100 × (T – T0) / (C – T0) = 50, where T is the optical density of the target compound after 48 h exposure. T0 is the optical density at time 0 and C is the control optical density. TGI represents the concentration of the target compound where 100 × (T – T0) / (C – T0) = 0 and it is the cytostatic effect. LC_50_ is the concentration of the target compound where 100 × (T – T0) / T0 = –50. LC_50_ also signifies the cytostatic effect and the control optical density is not used in the calculation.

## 3. Experimental

### 3.1. General

Unless otherwise stated, all chemicals and reagents were purchased from Sigma-Aldrich Chemical Co. Melting points were determined on an Electrothermal MEL-TEMP apparatus and are uncorrected. ^1^H-NMR and ^13^C-NMR spectra were recorded in DMSO-d_6 _on a Bruker 500 MHz instrument and the chemical shift (δ) values are reported in parts per million (ppm)relative to TMS. A Thermo Scientific LTQ Linear Trap LC/MS/MS system was used for mass spectrometry. UV spectra were recorded on a Beckman-Coulter DU-800 spectrophotometer. Analytical TLC was carried out on Sigma-Aldrich (cat # Z122785-25EA), 0.2 mm percolated silica gel polyester sheets with UV indicator. Elemental analysis was carried out by M-H-W Laboratories, Phoenix, AZ. Analysis of C, H, N were within ± 0.4% of theoretical values. The carbon numbering was shown for representative monomer **20a** and for one representative dimer **20b**. All others were referred similarly on the ^13^C-NMR assignments. 

### 3.2. General Procedure-A for N_9_-[(Z)-4'-chloro-2'-butenyl-1'-yl]-6-methoxypurine (**6**)

*Synthesis of 6-methoxypurine:* To a stirred suspension of 6-chloropurine (5.0 g, 32 mmol) in anhydrous methanol (240 mL) was slowly added 30 W% sodium methoxide in methanol (17.3 g, 320 mmol) at room temperature and then the reaction mixture was refluxed for 18 h. It was cooled to room temperature, neutralized with glacial acetic acid to pH 7.5–8.0 and then evaporated in a rotary evaporator to remove the solvent. The residue was treated with cold water (5 °C) (100 mL), the resulting solid was filtered, thoroughly washed with DI water. The product was crystallized from methanol as brownish white solid (3.5 g), 72% yield. ^1^H-NMR: δ 13.39 (1H, br s, NH), 8.50 (1H, s, H-8), 4.10 (3H, s, OCH_3_).

To a suspension of 6-methoxypurine (1.51 g, 10 mmol), anhydrous potassium carbonate (2.1 g, 15 mmol) in DMF (50 mL) at room temperature, was added *cis*-1,4-dichloro-2-butene (1.25 g, 10 mmol) and the contents stirred at room temperature for 5 h. The reaction mixture was filtered to remove the potassium carbonate that was also washed with DMF (25 mL). The filtrate and washings combined and evaporated under vacuum. The residue was chromatographed on a column of silica gel and the product was eluted with ethyl acetate: hexane 1:1 v/v. Evaporation of the homogeneous fractions resulted in a residue that was further crystallized from ethyl acetate-hexane as cream white rosettes, (1.3 g), 54% yield, m.p. 104–106 °C. ^1^H-NMR: δ 8.54 (1H, s, H-2), 8.36 (1H, s, H-8), 5.89–5.84 (2H, m, HC=CH), 5.4 (2H, s, N-*CH_2_*), 4.10 (3H, s, OCH_3_), 2.71 (2H, m, *CH_2_*Cl), 1.10 (3H, t, *J* = 7.5 Hz, OCH_3_). ^13^C-NMR: δ 160.22 (C-6), 151.76 (C-4), 151.49 (C-2), 129.66 (C-3'), 128.11 (C-2'), 120.51 (C-5), 53.84 (O*C*H_3_), 40.01 (C-1'), 39.85 (C-4'). Anal. Calcd. for C_10_H_11_N_4_OCl: C 50.32, H 4.65, N 23.47; Found: C 50.20, H 4.74, N 23.55.

*N_9_-[(Z)-4'-Chloro-2'-butenyl-1'-yl]-2,6-dichloropurine *(**5a**). A suspension of 2,6-dichloropurine (2.0 g, 11 mmol), anhydrous potassium carbonate (4.6 g, 34 mmol), 1,4-dichloro-2-butyne (2.1 g, 17 mmol) in anhydrous DMF was stirred at r.t. for 6 h. The reaction has been worked-up and purified on a column of silica gel as described in the general procedure-A , to give a major product **5a **and a minor dimeric product **5b**. Compound **5a **was isolated as a cream white solid (1.1g), 46% yield, m.p. 65–67 °C. ^1^H-NMR: δ 8.67 (1H, s, H-8), 5.93–5.91 (2H, m, HC=CH), 5.10–5.09 (2H, m, N-*CH_2_*), 4.54–4.52 (2H, m, *CH_2_*Cl). ^13^C-NMR: δ 153.23 (C-2), 150.95 (C-6), 149.59 (C-4), 148.0 (C-8), 130.44 (C-5), 130.24 (C-3'), 127.16 (C-2'), 40.53 (C-1'), 39.13 (C-4'). LC-MS (*m/z*): 277 [M+1]^+^, 100%. Anal. Calcd. forC_9_H_7_N_4_Cl_3_: C 38.95, H 2.54, N 20.19; Found: C 39.84, H 2.45, N 20.25.

*N_9_,N_9'' _-bis[(Z)-2'-Butenyl-1',4'-diyl]-2,6-dichloropurine *(**5b**). Compound **5b **was isolated in the above reaction as a brownish white solid (0.45g), 15% yield, m.p. 226–228 °C. ^1^H-NMR: δ 8.65 (2H, H-8, H-8''), 5.97 (m, 2H, HC=CH), 4.97 (4H, m, 2 × N-*CH_2_*). ^13^C-NMR: δ 153.33 (C-2, C-2''), 150.93 (C-6, C-6''), 149.60 (C-4, C-4''), 148.17 (C-8, C-8''), 130.52 (C-5, C-5''), 127.69 (C-2', C-3'), 40.95 (C-1', C-4'). LC-MS (*m/z*): 431 [M+1]^+^, 100%. Anal. Calcd. forC_14_H_8_N_8_Cl_4_: C 39.10, H 1.88, N 26.05; Found: C 39.0, H 2.0, N 25.95.

### 3.3. N_9_-[(Z)-4'-Chloro-2'-butenyl-1'-yl]-2-chloro-6-methoxypurine (**7**)

*Synthesis of 2-chloro-6-methoxypurine*: 2,6-dichloropurine (5.0 g, 27 mmol) was reacted with sodium methoxide (30 wt.% 14.6 g, 270 mmol) in anhydrous methanol (250 mL) under reflux for 16 h. The reaction has been worked-up as described above under general procedure-A The crude product was crystallized from methanol as snow white solid (4.0g), 82% yield. ^1^H-NMR: δ 13.52 (1H, br s, NH), 8.42 (1H, s, H-8), 4.1 (3H, s, OCH_3_).

2-Chloro-6-methoxypurine (1.9 g, 10 mmol), anhydrous potassium carbonate (2.1 g, 15 mmol), *cis-* 1,4-dichloro-2-butene (1.5 g, 12mmol) were stirred in DMF (50 mL) at room temperature for 4 h. The crude product was isolated as described under the general procedure-A. The product was chromatographed on a column of silica gel, eluent ethyl acetate/hexane 1:1 v/v. Cream white solid (1.8g) 64% yield, m.p. 136–138 °C. ^1^H-NMR: δ8.38 (1H, s, H-8), 5.9–5.83 (2H, m, HC=CH), 5.02 (2H, d, *J* = 5.5 Hz, N-CH_2_), 4.5 (2H, d, *J* = 5.5 Hz, CH_2_Cl), 4.1 (3H, s, OCH_3_). ^13^C-NMR: δ 160.73 (C-6), 152.94 (C-2), 151.32 (C-4), 144.04 (C-8), 129.96 (C-3'), 127.64 (C-2'), 119.77 (C-5), 54.87 (O*CH_3_*), 40.05 (C-1'), 39.15(C-4'). Anal. Calcd. for C_10_H_10_N_4_OCl_2_: C 43.93, H 3.69, N 20.51; Found: C 43.85, H 3.75, N 20.45.

*N_9_-[(E)-4'-Chloro-2'-butenyl-1'-yl]-2,6-dichloropurine *(**8a**). A suspension of 2,6-dichloropurine (2.0 g, 11 mmol), anhydrous potassium carbonate (4.6 g, 34 mmol), *trans*-1,4-dichloro-2-butene (2.15 g, 17 mmol) in anhydrous DMF was stirred at r.t. for 10 h. The reaction was worked-up as described in the general procedure-A. Chromatography of the resulting crude product on a column of silica gel yielded one major product **8a **and a minor dimeric product **8b.** Compound **8a **was isolated as a cream white solid (1.2 g), 40% yield, m.p. 70–72 °C. ^1^H-NMR: δ 8.78 (1H, H-8), 6.42 (1H, m, HC=CH), 5.80 (1H, m, HC=CH), 5.0 (2H, d, *J* = 5.5 Hz, N-*CH_2_*), 4.21 (2H, m, *CH_2_*Cl). LC-MS (*m/z*): 277 [M+1]^+^, 100%. Anal. Calcd. for C_9_H_7_N_4_Cl_3_: C 38.95, H 2.54, N 20.19; Found C 38.82, H 2.65, N 20.25.

*N_9_,N_9''_-bis[(E)-2'-Butenyl-1',4'-diyl]-2,6-dichloropurine *(**8b**). Pale brown solid (0.6g), 13% yield, m.p. 240 °C, decomposes. ^1^H-NMR: δ 8.77 (2H, s, H-8), 5.97 (2H, m, HC=CH), 4.96 (4H, 2 × N-*CH_2_*). LC-MS (*m/z*): 431 [M+1]^+^,100%. Anal. Calcd. for C_14_H_8_N_8_Cl_4_: C 39.10, H 1.88, N 26.05; Found: C 39.20, H 2.0, N 26.15.

*N_9_-*[4'-Chloro-2'-butynyl-1'-yl]*-6-chloropurine *(**9a**). A suspension of 6-chloropurine (2.5 g, 16 mmol), anhydrous potassium carbonate (3.5 g, 25 mmol) and 1,4-dichlorobutyne (3.0 g, 24 mmol) in DMF (150 mL) was stirred at room temperature for 3.0 h. The crude product was isolated as described in the general procedure-A. It was chromatographed on a column of silica gel using ethyl acetate/hexane 1:1 (v/v)as eluents to furnish **9a** as a brownish white solid, 2.3 g, 59% yield, m.p. 92–94 °C. ^1^H-NMR: δ 8.83 (1H, s, H-2), 8.77 (1H, s, H-8), 5.32 (2H, t, *J* = 2.0 Hz, N-*CH_2_*), 4.49 (2H, t, *J* = 2.0 Hz, *CH_2_*Cl). ^13^C-NMR: δ 151.79 (C-2), 151.35 (C-6), 149.23 (C-4), 146.74 (C-8), 130.72 (C-5), 80.76 (C-3'), 79.71 (C-2'), 33.43 (C-1'), 30.47 (C-4'). Anal. Calcd. for C_9_H_6_N_4_Cl_2_: C 44.84, H 2.51, N 23.24; Found: C 44.75, H 2.64, N 23.15. From the above column, the ethyl acetate/hexane 2:1 v/v and 100% ethyl acetate eluents furnished the minor dimeric product *N_9_,N_9'' _-bis*[2'-butynyl-1',4'-diyl]*-6-chloropurine *(**9b**) as a pale brown solid. 0.7 g, 12% yield, m.p. 220 °C decomposes. ^1^H-NMR: δ 8.95 (2H, s, H-2, H-2, H-2''), 8.75 (2H, s, H-8, H-8''), 5.24 (4H, 2 × N-*CH_2_*). ^13^C-NMR: δ 151.9 (C-2, C-2''), 151.5 (C-6,C-6''), 149.4 (C-4, C-4''), 146.9 (C-8, C-8''), 130.8 (C-5, C-5''), 80.5 (C-2', C-3'), 40.9 (C-1',C-4'). Anal. Calcd. for C_14_H_8_N_8_Cl_2_: C 46.82, H 2.25, N 31.20; Found: C 46.92, H 2.14, N 31.25.

*N_9_-[4'-Chloro-2'-butynyl-1'-yl]-2,6-dichloropurine *(**10a**). A suspension of 2,6-dichloropurine (1.9 g, 10 mmol), anhydrous K_2_CO_3_ (4.2 g, 30 mmol) and 1,4-dichloro-2-butyne (1.85 g, 15 mmol) was stirred in DMF at r.t. for 8 h under argon. The reaction has been worked-up as described in the general procedure-A. The resulting product was chromatographed on a column of silica gel tofurnish **10a **as the major product and **10b **as a minor dimeric product.Compound **10a **cream white solid (1.4 g) 50% yield, m.p. 80–82 °C. ^1^H-NMR: δ 8.79 (1H, s, H-8), 5.29 (2H, t, *J* = 2.0 Hz, N-*CH_2_*), 4.50 (2H, t, *J* = 2.0 Hz, *CH_2_*Cl). ^13^C-NMR: δ 152.87 (C-2), 151.23 (C-6), 149.91 (C-4), 147.66 (C-8), 130.41 (C-5), 81.11 (C-3'), 79.32 (C-2'), 33.66 (C-1'), 30.46 (C-4')). LC-MS (*m/z*): 275 [M+1]^+^, 100%.Anal. Calcd. for C_9_H_5_N_4_Cl_3_: C 39.23, H 1.83, N 20.33; Found: C 39.10, H 1.70, N 20.15.

*N_9, _N_9'' _-bis[2'-Butynyl-1',4'-diyl]-2,6-dichloropurine *(**10b**). It was isolated as a brownish white solid (0.6g), 14% yield, m.p. 210 °C decomposes. ^1^H-NMR: δ 8.78 (2H, s, H-8, H-8''), 5.25 (4H, s, 2 × N-*CH_2_*). LC-MS (*m/z*): 429 [M+1]^+^, 100%. Anal. Calcd. for C_14_H_6_N_8_Cl_4_: C 39.28, H 1.41, N 26.18; Found: C 39.12, H 1.55, N 26.25.

*N_9_-[4'-Chloro-2'-butynyl-1'-yl]-6-methoxypurine *(**11**). 6-Methoxypurine (1.0 g, 7.0 mmol), anhydrous potassium carbonate (1.6 g, 12.0 mmol), 1,4-dichlorobutyne (1.1 g, 9.0 mmol) was stirred at r.t. for 4 h. The crude product was isolated as described in the general procedure-A. Further purification on a column of silica gel using ethyl acetate/hexane 1:1 v/v as the eluent furnished a brownish white solid (1.1g), 70% yield, m.p. 141–143 °C. ^1^H-NMR: δ 8.57 (1H, s, H-2), 8.45 (1H, s, H-8), 5.25 (2H, t, *J* = 2.0 Hz, N-*CH_2_*), 4.48 (2H, t, *J* = 2.0 Hz, *CH_2_*Cl), 4.10 (3H, s, OCH_3_). ^13^C-NMR: δ 160.31 (C-6), 151.79 (C-2), 151.50 (C-4), 143.07 (C-8), 120.45 (C-5), 80.30 (C-2', C-3'), 53.95 (O*CH_3_*), 32.92 (C-1'), 30.52 (C-4'). Anal. Calcd. for C_10_H_9_N_4_OCl: C 50.75, H 3.83, N 23.67; Found: C 50.60, H 3.90, N 23.55.

*N_9_-[4'-chloro-2'-butynyl-1'-yl]-2-chloro-6-methoxypurine *(**12**). Brownish white solid, 61% yield, m.p. 143–145 °C. ^1^H-NMR: δ 8.48 (1H, s, H-8), 5.23 (2H, t, *J* = 2.0 Hz, N-*CH_2_*), 4.45 (2H, t, *J* = 2.0 Hz, *CH_2_*Cl), 4.11 (3H, s, OCH_3_). ^13^C-NMR: δ 160.85 (C-6), 152.64 (C-2), 151.61 (C-4), 143.82 (C-8), 119.75 (C-5), 80.66 (C-3'), 79.88 (C-2'), 54.98 (O*C*H_3_), 33.18 (C-1'), 30.50 (C-4'). Anal. Calcd. for C_10_H_8_N_4_OCl_2_: C 44.30, H 2.97, N 20.67; Found: C 44.15, H 3.15, N 20.55.

### 3.4.General procedure B for N_9_-[(E)-2',3'-dibromo)-4'-chloro-2'-butenyl-1'-yl]-6-chloropurine (**13**)

A suspension of **9a **(0.242 g, 1.0 mmol), pyridiniumtribromide (0.4 g, 1.3 mmol) in anhydrous dichloromethane (100 mL) was cooled to −10 °C while stirring. Anhydrous methanol (50 mL) was added drop wise during 15–20 minutes. The reaction mixture was allowed to warm-up and stirred for 20 h in a fume hood. The clarified reaction mixture was evaporated on a rotary evaporator at 30–35 °C without any quenching with sodium thiosulfate. The product was purified on a column of silica gel using ethyl acetate - light petroleum ether 1:1, 2:1 v/v as the eluents. It was further crystallized from ethyl acetate-light petroleum ether as a white crystalline solid 0.22 g, 54% yield, m.p. 88–90 °C. ^1^H-NMR: δ 8.80 (1H, s, H-2), 8.70 (1H, s, H-8), 5.40 (2H, s, N-*CH_2_*), 4.71 (2H, s, *CH_2_*Cl).^ 13^C-NMR: δ 151.70 (C-2), 151.30 (C-6), 149.0 (C-4), 146.7 (C-8), 130.70 (C-5), 121.30 (C-3'), 119.40 (C-2'), 50.40 (C-1'), 49.70 (C-4'). Anal. Calcd. for C_9_H_6_N_4_Br_2_Cl_2_: C 26.96, H 1.51, N 13.98; Found: C 26.80, H 1.65, N 14.15. The pyridinium salts were retained on the column.

*N_9_-[(E)-2',3'-Dibromo-4'-chloro-2'-butenyl-1'-yl]-6-methoxypurine *(**14**). Compound **11 **was used as the starting material and the above bromination procedure was followed. A crystalline white solid resulted, 65% yield, m.p. 127–129 °C. ^1^H-NMR: δ 8.56 (1H, s, H-2), 8.41 (1H, s, H-8), 5.45 (2H, s, N-*CH_2_*), 4.72 (2H, s, *CH_2_*Cl), 4.11 (3H, s, O*C*H_3_). ^13^C-NMR: δ 160.29 (C-6), 152.01 (C-4), 151.94 (C-2), 143.93 (C-8), 121.59 (C-5), 121.33 (C-3'), 119.46 (C-2'), 53.97 (O*C*H_3_), 50.42 (C-1'), 49.75 (C-4'). LC-MS (*m/z*): 397[M+1]^+^, 100%. Anal. Calcd. for C_10_H_9_N_4_OBr_2_Cl: C 30.29, H 2.29, N 14.13; Found: C 30.10, H 2.34, N 14.05.

*Synthesis of N_9_-(tetrahydropyran-2-yl)-6-chloropurine *(**25**). A suspension of 6-chloropurine (5.0 g, 32 mmol), 3,4-dihydro-2*H*-pyran (5.4 g, 64 mmol) and pyridinium-*p*-toluenesulfonate (PPTS) (3.3 g, 13 mmol) in anhydrous dichloromethane (250 mL) was stirred at room temperature in an atmosphere of argon for 64 h. The original suspension transformed in to a clear liquid and the starting material disappeared. The reaction mixture was evaporated and the residue was chromatographed on a column of silica gel. The THP ether was eluted with 100% ethyl acetate and that was isolated as a semi-solid. It solidified as a pale yellow soft solid upon cooling in a freezer (7.0 g), 91% yield. Homogeneous on TLC on silica gel plate, R_f_ 0.58, mobile phase 100% ethyl acetate. The starting material 6-chloropurine under the same conditions was slow moving, R_f_ 0.12. ^1^H-NMR: δ 8.92 (1H, s, H-2), 8.81 (1H, H-8), THP protons: 5.81–5.77 (1H, m), 4.01–3.99 (1H, m), 3.77–3.68 (1H, m), 2.37–2.28 (1H, m), 2.04–1.96 (2H, m), 2.04 (1H, m), 2.03 (2H, m).

### 3.5. General Procedure C for Synthesis of N_9_-(tetrahydropyran-2-yl)-6-(4-methoxyphenyl)purine (**26**)

*Suzuki-Miyaura cross coupling reaction*: A stirred suspension of 6-chloro-9-(tetrahydropyran-2-yl) purine **25**, (3.5 g, 15 mmol), 4-methoxyphenyl boronic acid (3.35 g, 22 mmol), anhydrous potassium carbonate (3.1 g, 22 mmol),tetrakis(triphenylphosphine)palladium (0), Pd (PPh_3_)_4_, (0.85 g, 0.74 mmol) in anhydrous toluene (150 mL) was gradually heated to 100 °C during 1 hr and then maintained at that temperature for 18 h. The TLC, silica gel plate, indicated the disappearance of the starting material R_f_ 0.45 and the formation of the new product R_f_ 0.54, mobile phase ethyl acetate-hexane 7:3 v/v. The reaction mixture was filtered while warm to remove the potassium and boron salts. The filtrate was concentrated and the resulting residue was crystallized from ethyl acetate-methanol 1:1 v/v as a brownish white solid (4.0g), 88% yield, m.p. 146–148 °C. ^1^H-NMR: δ 8.93 (1H, s, H-2), 8.87–8.82 (2H, m, Ar-H), 8.70 (1H, s, H-8), 7.18–7.13 (2H, m, Ar-H), 4.06–4.03 (1H, m, THP), 3.87 (3H, s,OCH_3_), 3.76–3.71 (1H, m, THP), 2.37–2.31 (1H, m, THP), 2.04–1.99 (2H, m, THP), 1.81–1.73 (1H, m, THP), 1.67–1.79 (2H, m, THP).

*Synthesis of 6-(4-Methoxyphenyl)purine *(**28**). To a suspension of compound **26** (3.2 g, 10 mmol) in methanol (100 mL) was added acetyl chloride (0.2 mL, 2.8 mmol) and the contents stirred overnight at room temperature for 24 h. The reaction mixture was evaporated, the residue was treated with water (100 mL) and the pH was adjusted to 7.5–8.0 with a saturated solution of sodium bicarbonate in water. The resulting solid was filtered, washed with DI water (100 mL) followed by cold ethanol (25 mL), hexane (25 mL) and dried under vacuum, brownish white solid (1.9 g), 81% yield. No further purification was necessary as the product was found to be homogeneous on silica gel TLC, R_f_ 0.3, mobile phase ethyl acetate-hexane 7:3 v/v. ^1^H-NMR: δ 8.97 (1H, s, H-2), 8.82–8.79 (2H, m, Ar-H), 8.75 (1H, s, H-8), 8.0 (1H, broad, NH), 7.2–7.17 (3H, m, Ar-H), 3.88 (3H, s, O*CH_3_*).

*N_9_-[(Z)-4'-Chloro-2'-butenyl-1'-yl]-6-(4-methoxyphenyl)purine *(**15a**). A suspension of 6-(4-methoxy-phenyl)purine (0.68 g, 3.0 mmol), anhydrous potassium carbonate (1.0 g, 7.2 mmol), *cis*-1,4-dichloro-2-butene (0.42 g, 3.4 mmol) was stirred at room temperature under argon atmosphere for 4h. The reaction mixture was worked-up as described in the general procedure-A. The resulting residue was chromatographed on a column of silica gel. Fractions 3–4 (100 mL) each from ethyl acetate-hexane (1:1) v/v furnished a brownish white solid **15a **(0.41 g), 43% yield, m.p. 129-131 °C. ^1^H-NMR: δ 8.92 (1H, s, H-2), 8.87–8.82 (2H, m, Ar-H), 8.61 (1H, s, H-8), 7.16–7.13 (2H, s, Ar-H), 5.92–5.89 (2H, m, HC=CH), 5.09–5.07 (2H, d, *J* = 9.0 Hz, N-*CH_2_*), 4.54–4.52 (2H, m, *CH_2_*Cl), 3.86 (3H, s, O*CH3*). ^13^C-NMR: δ 161.61 (C-13), 152.42 (C-4), 151.86 (C-6), 145.44 (C-8), 131.07 (C-11, C-15), 129.74 (C-3'), 129.62 (C-10), 128.08 (C-2'), 127.82 (C-5), 114.05 (C-12, C-14), 55.32 (O*C*H_3_), 39.68 (C-1'), 39.22 (C-4'). LC-MS (*m/z*): 315 [M+1]^+^, 100%. Anal. Calcd. for C_16_H_15_N_4_OCl: C 61.05, H 4.80, N 17.80; Found: C 61.10, H 4.92, N 17.90.

*N_9_,N_9''_-bis[(Z)-2'-Butenyl-1'4'-diyl]-6-(4-methoxyphenyl)purine *(**15b**). The fractions 5–8 from the above column chromatography yielded a brownish white solid (0.1 g), 11% yield, m.p. 163–165 °C. ^1^H-NMR: δ 8.93 (2H, s, H-2, H-2''), 8.89–8.84 (4H, m, Ar-H), 8.72 (2H, s, H-8, H-8''), 7.17–7.14 (4H, m, Ar-H), 5.99–5.96 (2H, m, HC=CH), 5.29–5.27 (4H, d, *J* = 10.0 Hz, 2 × N-*CH_2_*), 3.87 (6H, s, C-13-O*CH_3_*, C-13''-O*CH_3_*). ^13^C-NMR: δ 161.63 (C-13, C-13''), 152.45 (C-4, C-4''), 151.98 (C-6, C-6''), 151.73 (C-2, C-2''), 145.66 (C-8, C-8''), 131.10 (C-11, C-11'', C-15, C-15''), 129.70 (C-10, C-10''), 128.03 (C-2', C-3'), 127.85 (C-5, C-5''), 114.09 (C-12, C-12'', C-14, C-14''), 55.34 (C-13-O*C*H_3_, C-13''-O*C*H_3_), 40.20 (C-1', C-4'). LC-MS (*m/z*): 505 [M+1]^+^, 100%. Anal. Calcd. for C_28_H_24_N_8_O_2_: C 66.65, H 4.80, N 22.21; Found: C 66.52, H 4.92, N 22.33.

### 3.6. Synthesis of N_9_-(tetrahydropyran-2-yl)-6-(4-fluorophenyl)purine (**27**)

*Suzuki-Miyaura cross coupling reaction*: 9-(Tetrahydropyran-2-yl)-6-chloropurine (4.8 g, 20 mmol), 4-fluorophenyl boronic acid (4.2 g, 30 mmol), Pd(PPh_3_)_4_, (1.0 g, 0.9 mmol) in anhydrous dimethoxy-ethane (200 mL) was added a 2.7 molar saturated Na_2_CO_3_ solution in water (11.2 mL, 30 mmol). The reaction mixture was gradually heated to reflux during 2 h in an oil bath and then maintained at reflux for 7 h. Reaction was worked-up and purified as above to yield a brownish white solid (5.0 g), 83% yield. m.p. 146–148 °C. ^1^H-NMR: δ 9.0 (1H, s, H-2), 8.93–8.89 (2H, m, Ar-H), 8.88 (1H, s, H-8), 7.46–7.42 (2H, m, Ar-H), THP protons: 5.85–5.82 (1H, m), 4.06–4.03 (1H, m), 3.77–3.72 (1H, m), 2.38–2.35 (1H, m), 2.06–1.99 (2H, m), 1.84–1.72 (1H, m), 1.63–1.61 (2H, m).

*6-(4-Fluorophenyl)purine *(**29**). To a stirred suspension of 9-(tetrahydropyran-2-yl)-6-(4-fluorophenyl) purine (**27**, 3.75 g, 13 mmol) in methanol (200 mL) at room temperature was added acetyl chloride (0.4 mL, 5.6 mmol) and the reaction was worked-up as described in **26**. A brownish white solid resulted (2.6 g), 96% yield. The product was found homogeneous on TLC on a silica gel plate, R_f _0.3, mobile phase ethyl acetate-hexane (7:3) v/v. ^1^H-NMR: δ 8.94 (3H, s, H-2 and 2H Ar-H), 8.92 (1H, br, NH), 8.65 (1H, s, H-8), 7.45–7.40 (2H, m, Ar-H). 

*N_9_-[(Z)-4'-Chloro-2'-butenyl-1'-yl]-6-(4-fluorophenyl)purine *(**16a**). A suspension of 6-(4-fluorophenyl) purine **29** (0.65 g, 3.0 mmol), anhydrous potassium carbonate (1.0 g, 7.2 mmol), *cis*-1,4-dichloro-2-butene (0.413 g, 3.3 mmol) in DMF (50 mL) was stirred under argon at room temperature for 3.5 h. The reaction was worked-up as described in general procedureA.The crude reaction mixture contained a major product **16a **and a minor dimeric product **16b**. The above crude product upon crystallization from ethyl acetate-methanol furnished the dimeric product **16b **as feathery brownish white needeles. The mother liquor was concentrated and chromatographed on a column of silica gel using ethyl acetate-light petroleum ether (1:1, 2:1v/v)as the eluents that furnished a brownish white solid of **16a** (0.4 g), 43% yield, m.p. 94–96 °C. ^1^H-NMR: δ 8.99 (1H, s, H-2), 8.93–8.90 (2H, m, Ar-H), 8.67 (1H, 2, H-8), 7.45–7.41 (2H, m, Ar-H), 5.93–5.91 (2H, m, HC=CH), 5.10–5.09 (2H, d, *J* = 5.5 Hz, N-*CH_2_*), 4.54–4.52 (2H, m, *CH_2_*Cl).^ 13^C-NMR: δ 164.80 (d, ^1^*J*_CF _= 249 Hz, C-13), 152.13 (C-4), 151.75 (C-2), 151.39 (C-6), 146.09 (C-8), 131.85 (d, ^4^*J*_CF_ = 3.8 Hz, C-10), 131.75 (d, ^3^*J*_CF_ = 8.8 Hz, C-11, C-15), 129.99 (C-5), 129.82 (C-3'), 127.97 (C-2'), 115.73 (d, ^2^*J*_CF_ = 21.4 Hz, C-12, C-14), 39.74 (C-1'), 39.21 (C-4'). LC-MS (*m/z*): 303 [M+1]^+^, 10%, 267 [M+1]^+^-HCl, 100%. Anal. Calcd. for C_15_H_12_N_4_FCl: C 59.51, H 4.0, N 18.51; Found: C 59.40, H 4.10, N 18.45.

*N_9_,N_9''_-bis[(Z)-2'-Butenyl-1',4'-diyl]-6-(4-fluorophenyl)purine *(**16b**). 0.27 g, 18% yield, m.p. 198–200 °C. ^1^H-NMR: δ 8.99 (2H, s, H-2, H-2''), 8.94–8.92 (4H, m, Ar-H), 8.77 (2H, s, H-8,H-8''), 7.46–7.42 (4H, m, Ar-H), 6.01–5.99 (2H, m, HC=CH), 5.31–3.30 (4H, d, *J* = 5.5 Hz, 2 × N-*CH_2_*).^ 13^C-NMR: δ 164.83 (d, ^1^*J*_CF_ = 249 Hz, C-13, C-13''), 152.27 (C-4, C-4''), 151.76 (C-2, C-2''), 151.44 (C-6, C-6''), 146.34 (C-8, C-8''), 131.90 (d, ^4^*J*_CF_ = 3.8 Hz, C-10, C-10''), 131.79 (d, ^3^*J*_CF_ = 18.9 Hz, C-11, C-11'', C-15, C-15''), 130.09 (C-5, C-5''), 128.04 (C-2', C-3'), 115.80 (d, ^2^*J*_CF_ = 21.4 Hz, C-12, C-12'', C-14,C-14''), 40.29 (C-1', C-4'). LC-MS (*m/z*): 481 [M+1]^+^, 100%. Anal. Calcd. for C_26_H_18_N_8_F_2_: C 64.99, H 3.78, N 23.32; Found: C 65.10, H 3.82, N 23.45.

*N_9_-[(E)-4'-Chloro-2'-butenyl-1'-yl]-6-(4-methoxyphenyl)purine *(**17**). Cream white needles, 63% yield, m.p. 112–114 °C. ^1^H-NMR: δ 8.92 (1H, s, H-2), 8.87–8.82 (2H, m, Ar-H), 8.61 (1H, s, H-8), 7.16–7.13 (2H, s, Ar-H), 6.19–6.09 (1H, m, HC=CH), 5.83–5.73 (1H, m, HC=CH), 4.99–4.98 (2H, d, *J* = 9.0 Hz, N-*CH_2_*), 4.23–4.20 (2H, m, *CH_2_*Cl), 3.86 (3H, s, O*CH3*). ^13^C-NMR: δ 161.62 (C-13), 152.47 (C-4), 151.91 (C-6), 151.82 (C-2), 145.66 (C-8), 131.09 (C-11, C-15), 129.59 (C-10), 129.50 (C-3'), 129.02 (C-2'), 127.83 (C-5), 114.06 (C-12,14), 55.33 (C-13-O*C*H_3_), 44.08 (C-1'), 43.78 (C-4'). LC-MS (m/z): 277 [M+1]^+^, 100%. Anal. Calcd. for C_16_H_15_N_4_OCl: C 61.05, H 4.80, N 17.80; Found: C 61.15, H 4.95, N 17.95.

*N_9_-[(E)-4'-Chloro-2'-butenyl-1'-yl]-6-(4-fluorophenyl)purine *(**18a**). A suspension of 6-(4-fluoro-phenyl) purine (0.5 g, 2.3 mmol), anhydrous potassium carbonate (0.65 g, 4.7 mmol), *trans*-1,4-dichloro-2-butene (0.33 g, 2.6 mmol) in DMF (40 mL) was stirred at room temperature under argon for 22 h. The reaction has been worked-up as described in the general procedureA.The resulting product was chromatographed on a column of silica gel using ethyl acetate - light petroleumether (1:1) v/v as an eluent. Fractions of 100 mL were collected. The fractions 3-4 yielded **18a **as a cream white solid, homogeneous on silicagel TLC, mobile phase ethyl acetate-hexane 7:3 v/v, R_f_ 0.69 (0.4g), 57% yield, m.p. 73–75 °C. ^1^H-NMR: δ 8.98 (1H, s, H-2), 8.94–8.91 (2H, m, Ar-H), 8.68 (1H, s, C-8), 7.45–7.42 (2H, m, Ar-H), 6.16–6.12 (1H, m, HC=CH), 5.82–5.79 (1H, m, HC=CH), 5.01–5.0 (2H, d, *J* = 5.0 Hz, N-*CH_2_*), 4.22–4.20 (2H, m, *CH_2_*Cl).^ 13^C-NMR: δ 164.80 (d, ^1^*J*_CF _= 250 Hz, C-13), 152.16 (C-4), 151.80 (C-2), 151.43 (C-6),146.27 (C-8), 131.85 (d, ^4^*J*_CF_ = 2.52 Hz, C-10), 131.75 (d, ^3^*J*_CF_ = 8.8 Hz, C-11, C-15),129.96 (C-5), 129.59 (C-3'), 128.87 (C-2'),115.71 (d, ^2^*J*_CF_ = 21.4 Hz, C-12, C-14), 44.04 (C-1'), 43.85(C-4'). LC-MS (*m/z*): 303 [M+1]^+^, 100%. Anal. Calcd. for C_15_H_12_N_4_FCl: C 59.51, H 4.0, N 18.51; Found: C 59.25, H 4.16, N 18.65.

*N_9_,N_9''_-bis[(E)-2'-Butenyl-1',4'-diyl]-6-(4-fluorophenyl)purine *(**18b**). From the above chromatography, fractions 6–10 yielded this dimer **18b**, R_f _0.1, brownish white solid, (0.15 g), 13% yield, m.p. 217–219 °C. ^1^H-NMR: δ 8.95 (2H, s, H-2, H-2''), 8.93–8.90 (4H, m, Ar-H), 8.65 (2H, s, H-8, H-8''), 7.46–7.42 (4H, m, Ar-H), 5.98 (2H, m, HC=CH), 4.97–4.96 (4H, m, 2 × N-*CH2*). ^13^C-NMR: δ 164.86 (d, ^1^*J*_CF _= 252 Hz, C-13, C-13''), 152.61 (C-4, C-4''), 151.75 (C-6, C-6''), 151.61 (C-2, C-2''), 144.04 (C-8, C-8''), 131.44 (d, ^3^*J*_CF_ = 8.8 Hz, C-11, C-11'', C-15, C-15''), 131.32 (d, ^4^*J*_CF_ = 2.52 Hz, C-10, C-10''), 130.06 (C-5, C5''), 127.90 (C-2', C-3'), 115.06 (d, ^2^*J*_CF_ = 21.4 Hz, C-12, C-12'' C-14, C-14''), 43.88 (C-1', C-4'). LC-MS (*m/z*): 481 [M+1]^+^, 100%. Anal. Calcd. for C_26_H_18_N_8_F_2_: C 64.99, H 3.78, N 23.32; Found: C 64.82, H 3.85, N 23.45.

*N_9_-[4'-Chloro-2'-butynyl-1'-yl]-6-(4-methoxyphenyl)purine *(**19**). White solid, 80% yield, m.p. 119–121 °C. ^1^H-NMR: δ 8.93 (1H, s, H-2), 8.87–8.82 (2H, m, Ar-H), 8.70 (1H, s, H-8), 7.18–7.13 (2H, m, Ar-H), 5.31 (2H, t, *J* = 3.5 Hz, N-*CH_2_*), 4.50 (2H, m, CH_2_Cl), 3.87 (3H, s, OCH_3_). ^13^C-NMR: δ 161.70 (C-13), 152.67 (C-4), 152.0 (C-2), 151.58 (C-6), 145.19 (C-8), 131.13 (C-11, C-15), 129.49 (C-10), 127.67 (C-5), 114.11 (C-12, C-14), 80.34 (C-3'), 80.28 (C-2'), 55.34 (O*C*H_3_), 32.77 (C-1'), 30.55 (C-4'). LC-MS (*m/z*): 313 [M+1]^+^, 100%. Anal. Calcd. for C_16_H_13_N_4_OCl: C 61.44, H 4.19, N 17.91; Found: C 61.35, H 4.30, N 17.85.

*N_9_-[4'-Chloro-2'-butynyl-1'-yl]-6-(4-fluorophenyl)purine *(**20a**). Abrownish white solid, 64% yield, m.p. 128–130 °C. ^1^H-NMR: δ 9.02 (1H, s, H-2), 8.92–8.89 (2H, m, Ar-H), 8.75 (1H, s, H-8), 7.46–7.42 (2H, m, Ar-H), 5.33 (2H, t, *J* = 2.0 Hz, N-*CH_2_*), 4.50 (2H, t, *J* = 2.0 Hz, *CH_2_*Cl). ^13^C-NMR: δ 164.86 (d, ^1^*J*_CF_ = 249 Hz, C-13), 151.99 (C-2), 151.83 (C-4), 151.65 (C-6), 145.80 (C-8), 131.79 (d, ^3^*J*_CF_ = 8.8 Hz, C-11, C15), 131.68 (d, ^4^*J*_CF_ = 2.52 Hz, C-10), 129.87 (C-5), 115.75 (d, ^2^*J*_CF_ = 21.4 Hz, C-12, C-14), 80.43 (C-3'), 80.14 (C-2'), 32.86 (C-1'), 30.52 (C-4'). LC-MS (*m/z*): 301 [M+1]^+^, 100%. Anal. Calcd. for C_15_H_10_N_4_FCl: C 59.91, H 3.35, N 18.63; Found: C 60.0, H 3.40, N 18.55.

*N_9_,N_9''_-[2'-butynyl-1',4'-diyl]-6-(4-fluorophenyl)purine *(**20b**). A light brown solid, 11% yield, m.p. 225–227 °C. ^1^H-NMR: δ 8.99 (2H, s, H-2, H-2''), 8.93–8.90 (4H, m, Ar-H), 8.76 (2H, s, H-8, H-8''), 7.47–7.43 (4H, m, Ar-H), 6.01–5.30 (4H, s, 2 × N-*CH_2_*). LC-MS (*m/z*): 479 [M+1]^+^, 100%.Anal. Calcd. for C_26_H_16_N_8_F_2_: C 65.27, H 3.37, N 23.42; Found: C 65.15, H 3.45, N 23.55.

## 4. Conclusions

On the purine base selection, a strong electron withdrawing 2,6-dichloro system on the purine base is helpful for the cytotoxicity, ex: compounds **5a**, **5b**, **8b**, **10a**, **10b**. This also suggests that 2,6-dichloropurine base has significant potential for further work on the synthesis of anti-cancer compounds. The simultaneous electron withdrawing chlorine at the 2-position and electron donating methoxy group at 6-position on the purine ring did not contribute to the cytotoxic activity of the examples studied, compounds **7**, **12**. A 6-methoxy group on the purine base did not contribute to the cytotoxic activity in compounds **6** or **11**, however, a 6-methoxypurine bearing a chloromethyl vinylic dibromide moiety (compound **14**) exhibited excellent cytotoxic activity. This is the first report of activity in such a molecule and hence has a potential for further exploration in this direction. The 6-(4-methoxyphenyl)purine moiety elicited very potent activity (compound **19**). A riboside of this base also exhibited excellent cytostatic activity [10]. The consistency of this purine base in eliciting tumor inhibiting properties for wide range of cell lines is a very important finding from our work and from that reported in [10]. It is important to note that in our case the linker is an acyclic unsaturated butyne with a chloromethyl group and in the other case [10] it is a natural ribose sugar. Hence this purine base may be considered as potential candidate for future anti-cancer drug development work. The 6-(4-fluorophenyl)purine unit did not elicit any significant cytotoxicity in the compounds studied (**16a**, **20a**, **20b**). This suggests electron withdrawing 4-fluoro group on the phenyl ring was not helpful, a striking difference with the beneficial 4-methoxy group. 

In the linker selection, the methylchloromethylbutyne linker emerged as a viable non-sugar unsaturated linker (compounds **10a**, **19**). Even the dimeric compound with the butyne linker, **10b** exhibited cytotoxicity indicating this linker with the triple bond is important. Similarly the methylchloromethyl-*cis*-butyne linker compound **5a**, its dimer **5b **demonstrated excellent cytotoxic activity. The methylchloromethyl-*trans*-butene linker compound **17** and the dimer **8b** elicited good cytotoxic activity, but in other cases **8a **did not contribute any activity. The mechanism of action although not determined yet for our molecules may be different from purine nucleosides with natural sugars. Further useful structural modifications of the active purines **5a**, **5b**, **14 **and **19 **is in progress.
